# Homologous vs. homocratic neurons: revisiting complex evolutionary trajectories

**DOI:** 10.3389/fcell.2023.1336093

**Published:** 2023-12-21

**Authors:** Leonid L. Moroz, Daria Y. Romanova

**Affiliations:** ^1^ Department of Neuroscience and McKnight Brain Institute, University of Florida, Gainesville, FL, United States; ^2^ Whitney Laboratory for Marine Bioscience, University of Florida, St. Augustine, FL, United States; ^3^ Institute of Higher Nervous Activity and Neurophysiology of RAS, Moscow, Russia

**Keywords:** homology, nervous system evolution, Ctenophora, Mollusca, homoplasy, neurotransmitters, scRNA-seq, cell types

## Introduction

The diversity of neural systems across 34 animal phyla is astonishing (Bullock and Horridge, 1965; Schmidt-Rhaesa et al., 2015), with no recognized neuronal homologies among three basal eumetazoan lineages: Ctenophora, Cnidaria, and Bilateria. The remarkable molecular heterogeneity of neurons led to the hypothesis of independent origins of neurons in these lineages (Moroz, 2009; 2012). Genomic and phylogenetic data provided the initial evidence that neurons might evolve more than once (Moroz et al., 2014; Moroz and Kohn, 2016) or even three times (Moroz et al., 2021b) as a result of convergent evolution from the last nerveless ancestor of all Metazoa. To the best of our knowledge, the single origin of neurons is not supported by existing data, and this historically broadly accepted scenario should be critically evaluated as any other hypothesis. However, identifying deep, hierarchically complex, and distant homologies across phyla, especially at the level of specific cells or neuronal populations, is a highly controversial topic with no established criteria. Here, we provide a brief historical overview of the homology concept and then will discuss its applications to diverse nervous systems of invertebrates targeting the level of individual functionally characterized neurons, controlling specific behaviors.

## Brief history of ‘homology’

Richard Owen introduced the term *homology* (“homologue”) in 1843 to identify the same organ in various anatomical contexts of vertebrates within his concept of an archetype (Owen, 1843): “*the same organ in different animals under every variety of form and function*” (Owen, 1843). After Darwin, the concept of homology was transformed into the *evolutionary hypothesis* to retrace histories (=*genealogies*) of different structures. Homologous structures share a common ancestry, similar to the single origin of animals or green plants from their last common ancestors.

The concept was further *genealogically* developed by Ray Lankester in 1870, who tried to replace the term of homology with a more mechanistic name, ‘homogeny’ [avoiding the idealistic meaning of “ology” - see (Gouvêa and Brigandt, 2023)]. “*Structures which are genetically related, in so far as they have a single representative in a common ancestor, may be called homogenous*”—(Lankester, 1870). The name ‘homogeny’ did not survive in scientific literature. Lankester also coined the term ***homoplasy***: the similarity of traits *not due* to common ancestry (see historical summary in (Gouvêa and Brigandt, 2023). Thus, homoplasy is the alternative scenario to the ‘homology’ hypothesis–non-homology - implying convergent evolution.

**FIGURE 1:**
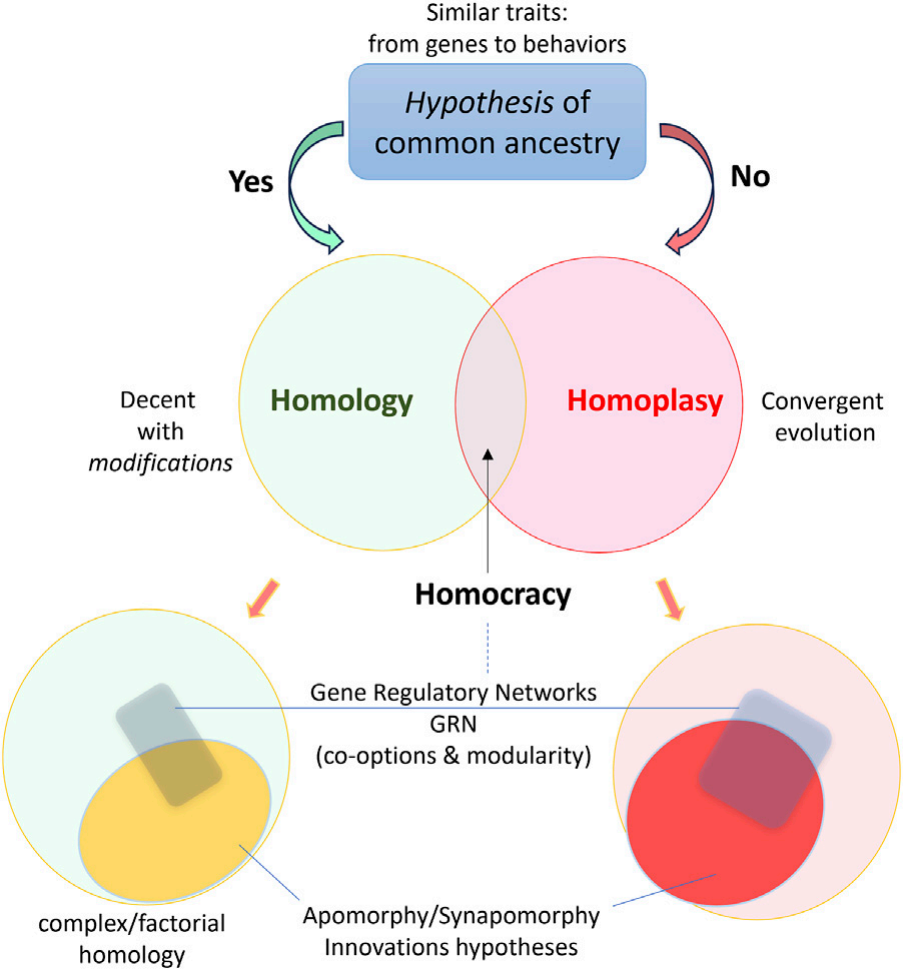
Homology as a hypothesis of evolutionary history with a simplified integration of terms reflecting the complex hierarchical modular organization of biological innovations.

Ernst May summarized the century of evolutionary thinking: “After 1859, there has been only one definition of homologous than makes biological sense a feature [character, structure and so on] is homologous in two or more taxa if it can be traced back to [or *derived from*] the same [a corresponding] feature in the presumptive common ancestor of these taxa” (Mayr, 1982). Today, hypotheses of homology have been broadly applied to all levels of biological organizations, from genes, proteins, and organelles to organs and organ systems both in adult organisms and in development (Wagner, 2014; 2016; DiFrisco et al., 2023a; DiFrisco et al., 2023b; Minelli, 2023; Rusin, 2023; Schlosser, 2023; Wanninger, 2024). However, many challenges exist at intermediate levels, especially for neuronal types and tissues, often associated with little-understood hierarchies of the so-called factorial concept of homologies (Minelli and Fusco, 2013). Figure 1 represents some terms related to the homology concept and biological innovations.

## Genealogy of neurons

Dmitry A. Sakharov provided the first application of homology to single individually identified neurons in 1970–1974 (Sakharov, 1970; 1972; 1974a; b). Specifically, he took advantage of large, sometimes truly giant, neurons (100–1000 microns in diameter) in the euthyneuran molluscs. These neurons are perfect models for neuroscience, enabling fundamental breakthroughs in deciphering cellular bases of behaviors, learning, and memory mechanisms (Kandel, 2001). The most illustrative examples of reference species are *Aplysia californica*, *Clione limacina*, *Lymnaea stagnalis*, *Helix pomatia*, *Pleurobranchaea californica*, *Tritonia diomedia,* and many others (Chase, 2002). Spherical neurons in these species are located on the ganglionic surface; they have different colorations and are perfectly visible and accessible for experimental manipulations (Kandel, 2001; Moroz et al., 2006; Moroz, 2011). In many cases, neurons have been functionally identified as part of neural circuits controlling stereotyped or learned behaviors (Kandel, 1976; 1979; Chase, 2002). Therefore, they are ideal paradigms for studying genealogies of neurons at the single-cell levels.

By mapping euthyneuran neurons with electrophysiological and, most importantly, neurotransmitter phenotypes, it was instantly recognized that nearly all neurons are remarkably different. Notably, these discoveries occurred in the 1970s, well ahead of time, and more than 50 years before the advent of single-cell genomics, so popular today. Euthyneuran neurons are individually unique and highly diverse in nearly all molecular and phenotypic characteristics. A surprising feature of this heterogeneity was the unprecedented diversity of their signal molecules, particularly small secretory peptides (with the early realization that most neurons are peptidergic, too). The unique combinatorics of diverse classical transmitters and neuropeptides in neural circuits were the hallmark of any studied system.

This situation raised a question: why are neurons so different; and, more specifically, why do neurons have different transmitters? Other researchers asked this question. Indeed, the logic was as follows. Suppose the neurotransmitter action is localized only within the space of the synaptic cleft (justifying this signal molecule’s very name as a just messenger = transmitter). Two transmitters (i.e., inhibitory and excitatory) might be sufficient in that case. In the most reductionistic viewpoint, even one transmitter might be adequate, considering the presence of different inhibitory and excitatory postsynaptic receptors. Yet, by the 1970–1980s, neuroscientists identified more than dozens of transmitters across every studied neural system *regardless* of the species’ phylogenetic position.

Sakharov hypothesized that neurons are different in their transmitter specificity because they are derived from genetically and phylogenetically different cell lineages (in modern terms). This proposal eventually led to a more generalized hypothesis that neurons might have different genealogies or ancestry (Sakharov, 1974a; b). The corollary of this *Neuronal Polyphyly* idea is that neurons evolved from different cell types, preserving their secretory/transmitter specificity at large evolutionary distances (Moroz, 2021). However, it was, and it is still challenging to determine the scope of such evolutionary distances and geological time of speciation events. In practical terms, Sakharov started identifying homologous individual neurons across model gastropod species with large, identified neurons and their transmitters to address these questions. Here, the classical criteria for homology were applied to the single-cell level for the first time (Sakharov, 1974b; 1976).

Three original Remanne criteria for homology were implemented (Remane, 1952):

1)The criterion of **position** and localization of target neurons within homologous part of molluscan ganglia (well-studied by that time) as well as branching patterns of neuronal processes;
  2)The criterion of **special quality**, primarily the transmitter/secretory phenotype, and in part, electrophysiology of identified neurons; both parameters are critical if the positional homology is not established and a neuron could migrate to other location [ganglion] in different species;
3)The criterion of **continuity**–i.e., the presence and subsequent identification of intermediate forms in related species, enabling the genealogy tracing within a given lineage by uniting the first and the second criteria. This assignment also addresses the tempo of evolution within a given lineage of animals.

Notably, these criteria emphasize relatively shorter evolutionary or taxonomical distances. Applying them for cross-phyla comparisons is challenging due to the lack of continuity for many phylum-specific traits and regionalization across diffuse and centralized neural systems. Other ‘complicated cases’ might include ultra-rapid changes in neuronal phenotypes and underlying gene regulation within specific animal classes and orders, coupled with the rise of novel behaviors; exploring novel habitats or food sources results in significant reorganization of neural circuits.

The initial quest to find homologous neurons was very successful, and several illustrative examples, as a proof-of-the concept study, are listed below. These findings are highly informative and hold their significance 50 years later. The first example is a pair of serotonergic modulator neurons known as metacerebral cells (MCC) controlling the initiation of the feeding program across all studied euthyneuran molluscs (Sakharov, 1974b; 1976; Weiss and Kupfermann, 1976; Gillette and Davis, 1977; Sudlow et al., 1998). This pair of cells is also the record example of the best-traced single-cell homology back to the last common ancestor of this lineage more than 380 million years ago (Kumar et al., 2022). The other examples included neurosecretory cells controlling osmotic homeostasis, such as single R15 in *Aplysia* and their homologs in pulmonate molluscs; and clusters of *Aplysia* R3-13 and LYC/D neurons in pulmonates (Sakharov, 1974b; 1976; Moroz, 1985) with perhaps similar functions. Later, other neuronal homologs were identified in feeding and locomotory circuits, center pattern generators, as well as neurosecretory cells controlling egg-laying (Kupfermann, 1970). These discoveries opened an exciting opportunity for novel evolutionary classification of neurons toward a natural system of neurons across taxa (Moroz, 2018). Regrettably, the euthyneuran molluscs and their neural systems are often forgotten in the recent literature about cell type evolution. In part, the outlined neuronal polyphyly hypothesis could explain (via multiple parallel genealogies of transmitter secretory phenotypes) why different neurons within circuits have different transmitters and why the same transmitters are present and conserved in various neural systems.

However, by tracing the homologies of serotonergic and dopaminergic neurons, it was noticed that serotonin (5-HT)-containing neurons are evolutionary more conservative in their positions, numbers, and functions than evolutionary, more dynamic dopaminergic neurons (Moroz, 1991). It led to studying homologous behaviors across molluscs and beyond, recognizing that 5-HT neurons control, modulate, and integrate specific behaviors associated with general arousal components in locomotion, feeding, stress reactions, etc. (Sakharov, 1990; Moroz, 1991; Gillette et al., 2000; Gillette, 2006; Lee et al., 2023) with the broadest possible innervation of peripheral organs (Moroz et al., 1997). In contrast, dopamine/catecholamines control more specific components of feeding and respiratory programs within the same lineages, implying the hierarchy of transmitters and underlying behaviors (Winlow et al., 1992). In this respect, Moroz suggested that the antioxidant properties of 5-HT contribute to the greater evolutionary conservation of serotoninergic neurons and functions, in contrast to dopaminergic and related catecholaminergic neurons, which can be easily oxidized, and their product might act as prooxidants.

Regardless of the causality, it was a surprising consensus to realize that 5-HT controls and integrates similar (as in molluscs, e.g. (Rosen et al., 1989)) types of arousal-associated behaviors across phyla (Sakharov, 1990), with the most illustrative examples in leeches (Lent, 1974; Lent, 1985; Lent et al., 1988), and with remarkably similar volume integration of effectors by a pair of 5HT-containing Retzius neurons in each segmental ganglion (Lent, 1985). There are numerous similar observations of 5HT-dependent control among representatives of bilaterians, including Arthropoda, Annelida, Nematoda, and Mollusca (Hay-Schmidt, 2000; Gillette, 2006; Tierney, 2020). Thus, at least some types of secretory-specific neurons might be involved in integrating *homologous behaviors*, but this statement might not be generalized for all transmitters, circuits, and species with high evolutionary plasticity at relatively small phylogenetic distances (e.g., across families). Here, we refer to animal lineages under substantial environmental pressure, such as adaptation to hypoxic conditions while exploring new habitats (e.g., freshwater pulmonate gastropods or meiofauna) or anthropogenic factors of catastrophic nature for entire ecosystems.

In summary, different transmitters and neurons secreting these transmitters might have different evolutionary dynamics. There are facts indicating a shift of transmitter phenotypes both in development within the same species (Demarque and Spitzer, 2012; Spitzer, 2015; Spitzer, 2017; Bertels et al., 2022) and macro-evolution (Bertuzzi et al., 2018; Moroz et al., 2021b). Macroevolutionary shifts might occur in arthropods, where glutamate is primarily localized in efferent neurons with motor function vs. the predominant presence of glutamatergic neurons in sensory and interneuron parts of molluscan and vertebrates’ nervous systems. Furthermore, the evolutionary expansion of glutamatergic neurons in vertebrate brains is very dramatic; it might be linked to higher bioenergetic demands, as summarized elsewhere (Moroz et al., 2021a).

Understandably, the examples mentioned above of mosaic distribution of glutamatergic and dopaminergic neurons across phyla do not always imply the homologization of these neuronal populations. In other words, a single or a few molecular markers or modules cannot be an unbiased criterion for neuronal homologization. The modular nature of gene regulatory networks (Wagner, 2014; DiFrisco et al., 2023a; DiFrisco et al., 2023b; Rusin, 2023) and lineage-specific evolutionary changes (apomorphies and synapomorphies) are not yet incorporated in the quest to identify homologous neurons across taxa.

## Complex homologies, cell-type tree of life

Introducing molecular biology into neuroscience dramatically increased mechanistic and genomic deciphering of neuronal functions. The emerging and growing complexity of neuronal populations led to identifying many proteins, genes and non-coding regions as regulators of neuronal phenotypes. The concepts of terminal selection of neuronal identity and transcriptional factor-specific barcoding of neuronal phenotype have been developed using an ultra-small number of neurons in the nematode *C. elegans* as a reference point (Hobert, 2016; 2021). Nevertheless, targeting homology assessments for single neuronal types across species is still challenging, with no universal criteria to define molecular reporters at large evolutionary distances (e.g., classes and phyla). Here, we also refer to molecular markers for practical applications (e.g., *in situ* hybridization or immunohistochemical experiments) to label neural populations in representatives of basal metazoan phyla with limited information about the neural organization.

Many researchers recognized that similar molecular markers and genes encoding master regulators expressed in non-homologous structures. Nielsen and Matrinez (2003) provided many illustrative examples in this direction, with surprises of expression of Distal-less and Pax 6 in unexpected places across ectoderm and mesoderm derivatives, which are not homologous embryonic layers (Nielsen and Martinez, 2003). But these data illuminate modular ways to encode more generalized ‘elementary’ molecular functions such as [photo]reception (Pax 6), making tissue “tips” (Dlx), holes (Bra), and that is the main reason that they can be co-opted in different structures (or cells).

To reflect this situation, Nielson and Martinez introduced the term *homocracy* (from ‘democracy’). “Structures are homocratic if they express the same patterning gene(s).” Homocratic structures might be homologous, but the same genes may have different cooption in non-homologous structures and molecular modules. This explanation means recruitments of similar genes in different programs, with many examples when complex structures might have chimeric origins, such as eyes and neural systems with mosaic expressions of numerous genes. On the other hand, “homologous structures are homocratic in many cases”; this descriptive terminology could be applicable to different neurons and neuronal cell types. The modular nature of genome regulation involves multiple distant non-coding cys-regulatory elements, which allows rapid co-option of many molecular blocks by ‘bringing’ them from one functional domain to another domain using small structural changes in the regulatory machinery [Hinman et al., 2003; Davidson, 2010). This type of molecular modular architecture has some functional redundancy. It could be a foundation (exaptation, see (Gould and Vrba, 1982)] for very rapid transitions from temporal to spatial differentiation outcomes and different cell phenotypes.

Following this inherently molecular modular and redundant architecture, it was clear that there are no pan-neuronal genes (Moroz and Kohn, 2015) as universal molecular markers to identify neurons across large evolutionary distances phenotypically.

It might be valuable to consider not only the cooption of different genes to different functions but also defining versatile functional modules as groups of proteins and genes co-expressed and co-regulated, which incorporate the concept of terminal selection of neuronal identity (Hobert, 2016; 2021) and modularity of neuronal machinery (Arendt, 2020). The idea of reconstruction of the metazoan Cell-type Tree of Life is an exciting and promising direction (Arendt et al., 2016). Here, the cell types are treated as biological species with different molecular regulatory modules and evolutionary trajectories, which overlap with earlier hypotheses of tracing neuronal homologies. The quantitative metric was introduced by employing tools of statistical geometry to evaluate the treeness models of cell type evolution (Kin et al., 2015; Liang et al., 2015). However, it was not applied to individual neurons or specific neuronal populations, and broad comparative single-cell genomic data are required from representative of *all* animal phyla (Moroz, 2018; Moroz and Romanova, 2022). The taxonomical level of resolution should *only empirically* come from the extensive development of comparative single-cell datasets combined with building cell trees (Arendt et al., 2016) across *all* 30+ animal phyla and *all* 90+ classes of Metazoa, perhaps even targeting specific orders and families as can be demanded from respective ecological contexts.

With new tools of comparative single-cell genomics on the horizon (Tarashansky et al., 2021), it is possible to provide homologization of cell type in sponges (Musser et al., 2021), where different scRNA-seq data allowed detection of cellular homologs at large evolutionary distances. Whether cell-type neuronal homologs can be found across phyla is still largely unknown. The *terra incognita* is the complexity, diversity of molecular modules, and highly dynamic regulation of cell phenotypes. Thus, returning to the classical continuity criterion would be reasonable by revisiting a well-established lineage of identified neurons within closely and more distantly related genera, families, orders, and subclasses (within gastropods, leeches, and arthropods) as proposed initially by Sakharov (Sakharov, 1974b). Such a strategy will systematically enable the deciphering of apomorphies, synaptomorphies, gene-regulatory networks, and homoplasy (Figure 1) within specific neuronal lineages, and in the context of their evolutionary connectivity and functions as a foundation for unbiased genealogy of neurons across taxa.
